# Suppression Subtractive Hybridization Analysis of Genes Regulated by Application of Exogenous Abscisic Acid in Pepper Plant (*Capsicum annuum* L.) Leaves under Chilling Stress

**DOI:** 10.1371/journal.pone.0066667

**Published:** 2013-06-18

**Authors:** Wei-Li Guo, Ru-Gang Chen, Zhen-Hui Gong, Yan-Xu Yin, Da-Wei Li

**Affiliations:** 1 College of Horticulture, Northwest A&F University, Yangling, Shaanxi, P. R. China; 2 State Key Laboratory of Crop Stress Biology in Arid Areas, Northwest A&F University, Yangling, Shaanxi, P. R. China; University of Delhi South Campus, India

## Abstract

Low temperature is one of the major factors limiting pepper (*Capsicum annuum* L.) production during winter and early spring in non-tropical regions. Application of exogenous abscisic acid (ABA) effectively alleviates the symptoms of chilling injury, such as wilting and formation of necrotic lesions on pepper leaves; however, the underlying molecular mechanism is not understood. The aim of this study was to identify genes that are differentially up- or downregulated in ABA-pretreated hot pepper seedlings incubated at 6°C for 48 h, using a suppression subtractive hybridization (SSH) method. A total of 235 high-quality ESTs were isolated, clustered and assembled into a collection of 73 unigenes including 18 contigs and 55 singletons. A total of 37 unigenes (50.68%) showed similarities to genes with known functions in the non-redundant database; the other 36 unigenes (49.32%) showed low similarities or unknown functions. Gene ontology analysis revealed that the 37 unigenes could be classified into nine functional categories. The expression profiles of 18 selected genes were analyzed using quantitative RT-PCR; the expression levels of 10 of these genes were at least two-fold higher in the ABA-pretreated seedlings under chilling stress than water-pretreated (control) plants under chilling stress. In contrast, the other eight genes were downregulated in ABA-pretreated seedlings under chilling stress, with expression levels that were one-third or less of the levels observed in control seedlings under chilling stress. These results suggest that ABA can positively and negatively regulate genes in pepper plants under chilling stress.

## Introduction

Pepper (*Capsicum annuum* L.) is a member of the *Solanaceae* family and an important vegetable and spice crop valued for its aroma, taste, pungency and flavor. The pepper fruit is the second most consumed vegetable worldwide [1] and an excellent source of many essential nutrients for humans, particularly vitamins, minerals and carotenoids. In addition, some pepper cultivars contain significant quantities of capsaicinoids, a group of pungent, phenolic-derived compounds with potent physiological and pharmacological properties [2]. Different types of peppers, including chili, mild and sweet peppers are cultivated worldwide. In China, over 1.5 million hectares of pepper are grown annually, and pepper is recognized as a high value-added crop [2]. The global demand for pepper fruit is increasing; therefore, the development of strategies to enhance its production and fruit quality through improved agricultural practices are required. As part of this effort, we are interested in investigating of plant defense mechanisms, in order to improve plant resistance to environmental stresses.

Low temperature is one of the most important abiotic factors limiting the growth, development and geographical distribution of plants [3]. Pepper plants originate from tropical regions and are very sensitive to low temperature. The optimal growth temperature for pepper plants ranges from 21°C to 27°C [4]. Pepper plants exposed to cold stress exhibit various symptoms, including flaccid stems and leaves, wilting and necrosis [4], [5]. Furthermore, low temperature affects the vegetative development and reproduction of the pepper by impairing the function of the female organs and reducing the number of viable pollen grains per flower [6]. Thus, the fruits obtained from plants cultivated under low night temperature (14°C or lower) are typically deformed and seedless, resulting in economic losses.

In response to low non-freezing temperature stress, a cascade of events are triggered, including changes in gene expression and the induction of biochemical and physiological modifications, resulting in enhanced tolerance of the plants to low temperature [7], [8]. External application of abscisic acid (ABA) mimics the effect of stress conditions and plays a critical role in this process [9], [10]. ABA, as a stress signal, enhances antioxidant defenses and reduces the accumulation of reactive oxygen species (ROS) in responses to abiotic stress, including low temperature [11], and drought [12]. In addition, ABA plays a protective role in the responses to abiotic stress through the regulation of genome-wide gene expression, especially in drought, high salt and cold treatments [13], [14]. Knight et al. [15] reported that ABA signaling mediates the adaptation to cold stress through inducing the expression of CBF (CBF1–3) transcription factors, which bind to C-repeat (CRT; dehydration-responsive) promoter elements to induce the expression of cold-regulated genes. Recently, there has been an increase in the number of studies investigating ABA-mediated gene expression in plants. Several reports have shown that ABA regulates gene expression mainly at the transcription level in *Arabidopsis* [16], [17]. Transcriptional analyses of ABA-responsive genes identified over 1350 genes that are either upregulated or downregulated in response to ABA [17]. Numerous studies employing microarray techniques have identified the ABA-responsive transcriptome in *Arabidopsis*, rice [13], [18], sorghum [19], and other plant species [20]. Although much of the knowledge concerning the ABA-mediated transcriptome has been derived from the aforementioned plant species, direct studies are required to identify ABA-regulated genes under abiotic stress in other cold-sensitive crops. Guo et al. [21] reported that exogenous application of ABA increased the tolerance of pepper seedlings to chilling-induced oxidative damage, mainly by enhancing the activity of antioxidant enzymes and expression of related genes; however, ABA-mediated gene regulation in response to chilling stress has not yet been fully characterized in pepper plants. Increased knowledge of the components of the pepper stress response may allow us to develop new strategies to improve pepper production by increasing stress tolerance.

Suppressive subtractive hybridization (SSH) is a powerful approach for identifying and isolating genes that are differentially expressed at low levels [22]. Compared with other techniques such as DNA microarray technology, which has been used to validate the results of SSH [23], the major advantage of SSH is the combination of normalization and subtraction, which can both identify abundant differentially expressed genes and enrich rare transcripts to facilitate the identification of novel genes [24]. Moreover, this technique circumvents the limitations of global gene expression analysis as it does not require complete genomic sequence information or microarrays; therefore, SSH can be used to study plants for which limited available sequence information is available [25]. Recently, SSH has been used to study plant responses to abiotic stress, with the ultimate aim of enhancing abiotic stress tolerance in plants [3], [25].

In the present study, RNA was isolated from the leaves of pepper plants exogenously pretreated with ABA or water (control) and then subjected to a time course of chilling stress (0 to 48 h), and used to generate forward and reverse SSH libraries. Genes which were up- or downregulated by ABA treatment were identified through homology searching of nucleotide sequences, and the expression of these genes was confirmed using quantitative PCR (qPCR). The identification and functional characterization of ABA-regulated genes associated with cold tolerance may provide valuable information for improving plant tolerance to chilling stress and harsh conditions.

## Results

### 2.1 ABA alleviates the visible symptoms of chilling injury in pepper leaves

ABA effectively reduced the visible symptoms of leaf damage in pepper seedlings subjected to chilling stress (Fig. S1). Wilting appeared after 1 h of chilling stress in control plants, while ABA-pretreated leaves did not exhibit withering until 12 h chilling stress. Stem drooping was observed in control seedlings after 6 h chilling stress; after 24 h, the stems started to recover their original appearance, and gradually, expanded leaves were observed after 48 h chilling stress, suggesting that the signs of recovery are likely to be associated with adaptive defense to chilling in the pepper.

### 2.2 Effects of ABA on electrolyte leakage, chlorophyll content, net photosynthetic rate and stomatal conductance in pepper leaves under chilling stress

**Figure 1:**
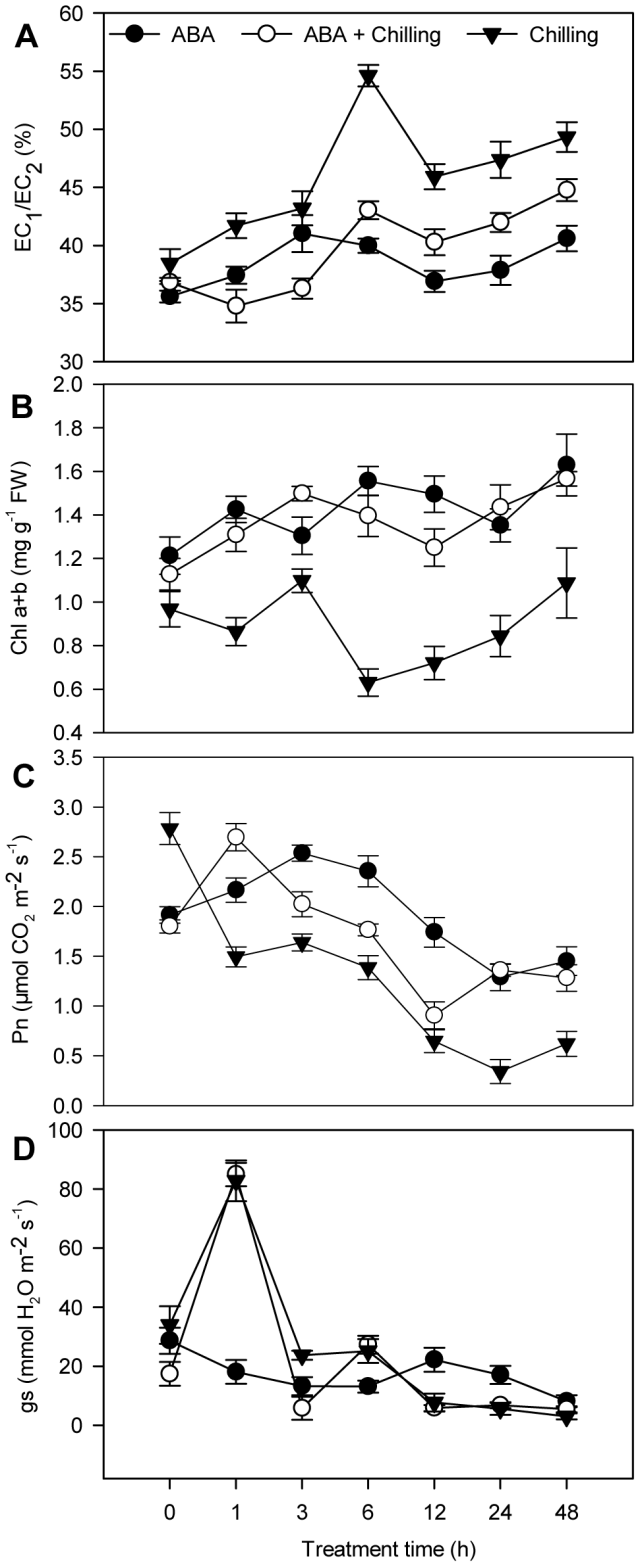
Effect of ABA application on EC1/EC2 (A), Chla+b (B), Pn (C) and g_s_ (D). 72 hours after application of 0.57 mM abscisic acid (ABA) by foliar spray, the plants were subjected to chilling stress at 6°C (day/night). Values are means ± SE (n = 4). Closed circle: ABA-pretreated samples under room temperature; open circle: ABA-sprayed samples prior to chilling stress; triangle down: water-sprayed samples prior to chilling stress.

Electrolyte leakage increased throughout the experimental period under chilling stress (Fig. 1A). Moreover, chilling led to significantly higher electrolyte leakage in the leaves of control plants than ABA-pretreated leaves. Chilling stress also induced a greater loss of chlorophyll pigment in control plants than in ABA-pretreated plants (Fig. 1B). The net photosynthetic rate in control chilling-stressed plants reduced rapidly until the end of the experimental period (Fig. 1C), whereas, application of ABA prior to chilling stress prevented the reduction in the photosynthetic rate triggered by chilling stress. Leaf stomatal conductance rapidly peaked after 1 h and abruptly decreased after 3 h chilling stress in both control and ABA-pretreated pepper seedlings (Fig. 1D). Meanwhile, the stomatal conductance of ABA-pretreated plants subjected to chilling stress was lower than control plants at 3 h, and thereafter basically similar to control plants, implying that chilling did not have an obviously different effect on stomatal behavior in control and ABA-pretreated plants after 6 h chilling stress. Taken together, these results suggest that exogenous application of ABA enhances the tolerance of pepper plants to chilling stress by protecting against increased electrolyte leakage from the cell membrane, the loss of chlorophyll pigment and reduced net photosynthetic rate.

### 2.3 Quality of the subtracted cDNA libraries

To identify differentially expressed genes in pepper seedlings pretreated with ABA under chilling stress, SSH was used to construct both forward and reverse subtractive cDNA libraries. The integrity and quality of the total RNA samples was assessed as described in Materials and Methods (Fig. S2). The quality of the mRNA purified from the total RNA samples was good, as the bulk of mRNA ranged in size from 250 to 2000 bp (Fig. S3). To estimate subtraction efficiency, the second-round PCR products were analyzed on 1.0% agarose ethidium bromide-stained gels (Fig. S4). The PCR fragments obtained were mainly between 300 and 750 bp in length (Fig. S5). Taken together, these results confirmed the generation of high-quality subtracted cDNA libraries.

### 2.4 Characterization of expressed sequence tags in the forward and reverse SSH libraries

In total, 438 clones from the forward SSH library and 330 clones from the reverse SSH library were confirmed through PCR amplification and selected for DNA sequencing. After removing the regions with low quality sequencing data, vector and adaptor sequences, and clones with sequences of less than 100 bp, 126 and 109 high-quality expressed sequence tags (ESTs) were obtained from the forward and reverse SSH libraries, respectively. The CAP3 assembly program was used to identify the ESTs representing redundant transcripts. The 235 high-quality ESTs were assembled into 18 contigs and 55 singlets, generating 73 assembled sequences. Of these, 40 sequences were upregulated after ABA treatment under chilling stress in the forward SSH library and 33 sequences were downregulated after ABA treatment under chilling stress in the reverse SSH library. These unigenes were submitted to GenBank dbEST (www.ncbi.nlm.nih.gov/dbEST) with the accession numbers JZ198744-JZ198816 (Table S1).

**Figure 2:**
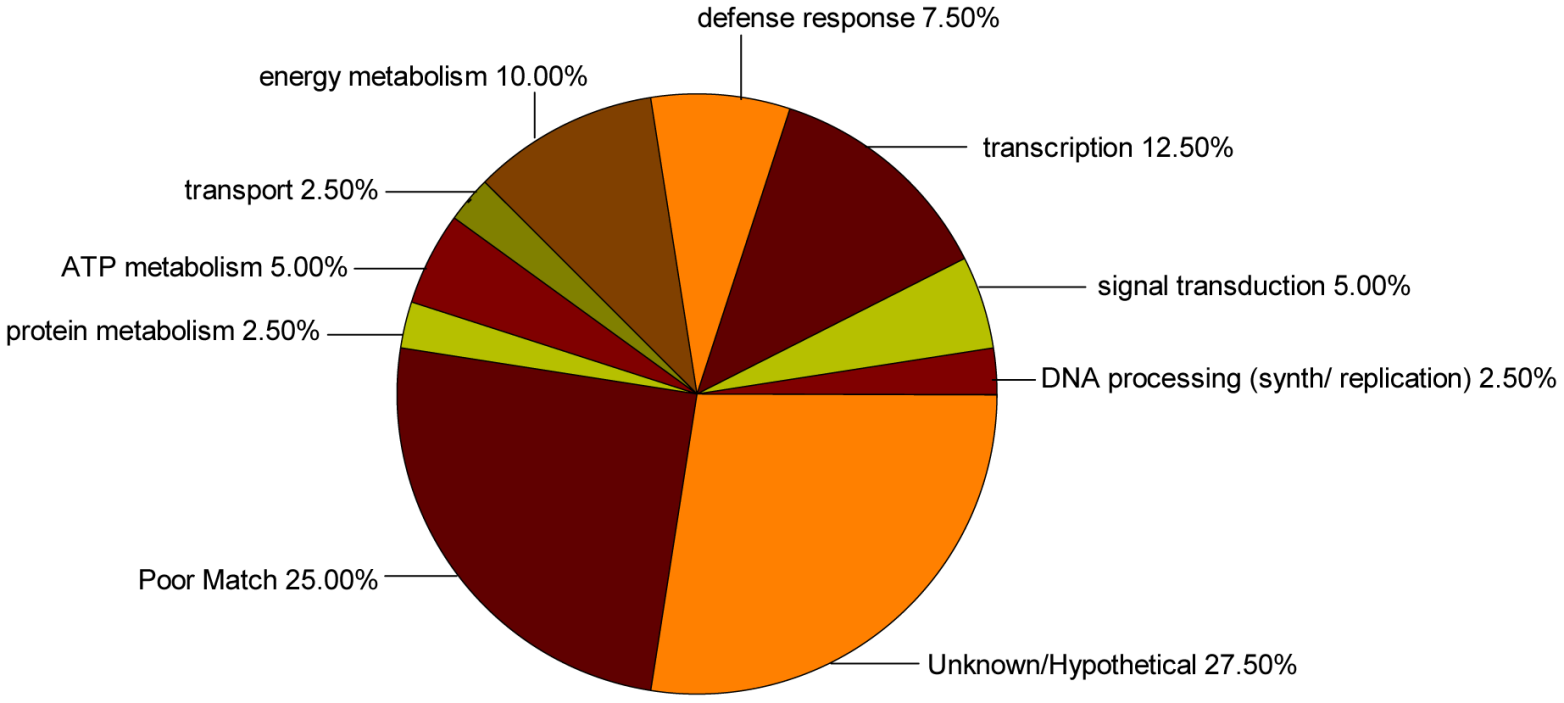
Functional classification of unigenes from the forward SSH library within biological process category.

**Figure 3:**
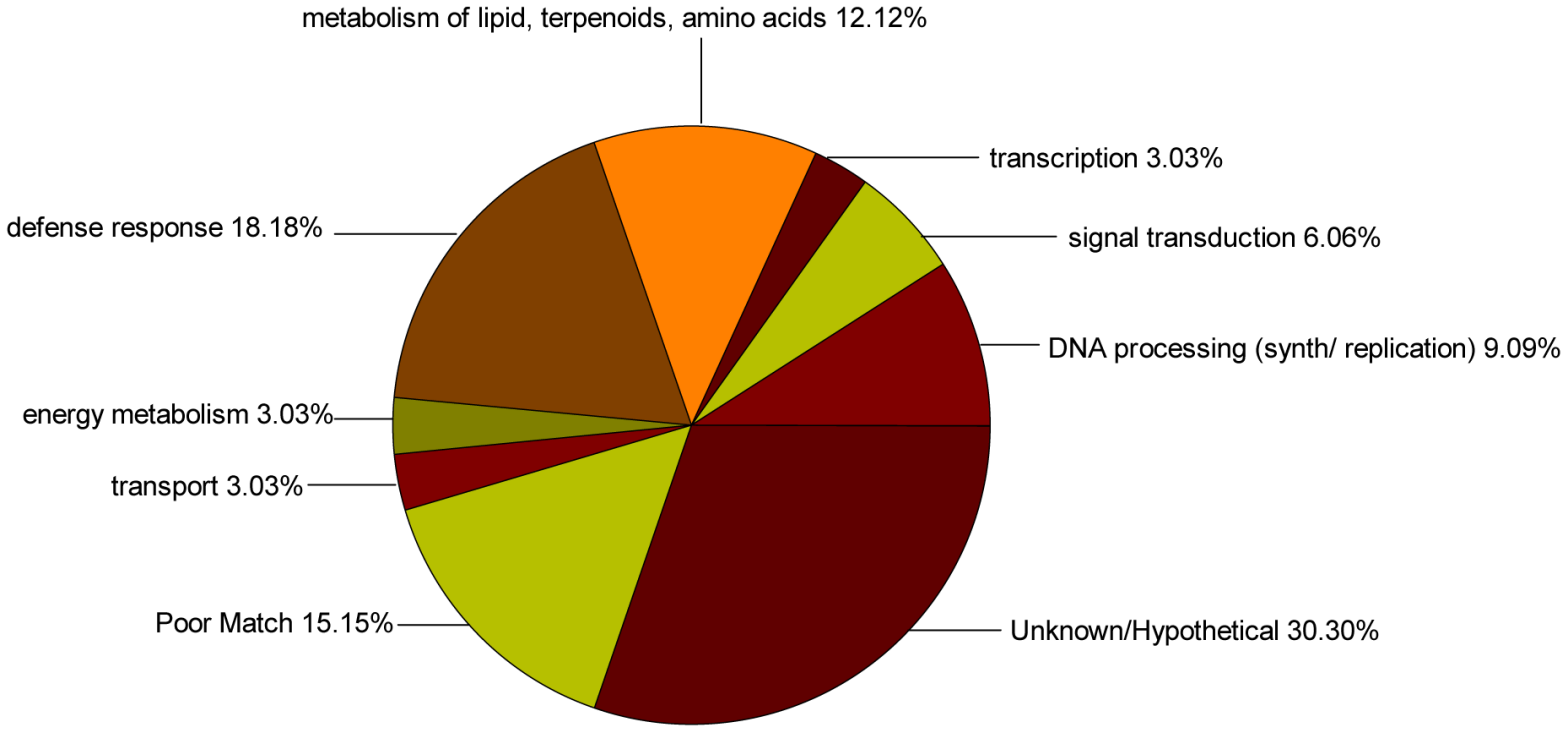
Functional classification of unigenes from the reverse SSH library within biological process category.

The sequences identified from the forward and reverse SSH libraries were analyzed using the blastx and blastn programs (with a cutoff *E*-value of 1e ≤1^-03^) and subsequently categorized into several putative functional categories according to gene ontology (GO) analysis. Ten (25.00%) unigenes from the forward SSH library and five (15.15%) unigenes from the reverse library had a low *E*-value (> 1e-03) or no matching sequences in the NCBI database; 11 (27.50%) unigenes from the forward SSH library and 10 (30.30%) unigenes from the reverse library were homologous to genes with unknown functions; and 19 (47.50%) of the 40 unigenes from the forward SSH library had a significant homology with genes of known functions, and were subsequently classified using GO annotations. Within the biological process category, the 19 unigenes were classified into eight primary functional categories, including transcription, signal transduction, defense response, transport, and energy metabolism (Fig. 2). The majority of genes were associated with transcription (12.5%), followed by energy metabolism (10.00%), defense response (7.50%), signal transduction (5.00%), and ATP metabolism (5.00%). In total, 18 (54.55%) of the 33 unigenes from the reverse SSH library had significant homology with genes of known functions and were divided into seven functional categories (Fig. 3), including defense response (18.18%), lipid, terpenoids, amino acids metabolism (12.12%), DNA processing (9.09%), signal transduction (6.06%), transcription (3.03%), energy metabolism (3.03%), and transport (3.03%).

### 2.5 Genes potentially involved in chilling tolerance

To gain further insight into the molecular mechanisms regulated by ABA-responsive genes in pepper plants under chilling stress, genes could be divided into six GO categories, including energy metabolism, defense response, signal transduction and transcription. These unigenes are presented in Table 1. The corresponding ESTs associated with the putative ABA-regulated genes related to chilling stress, such as alcohol dehydrogenase, superoxidase dismutase, plasma membrane calcium ATPase, and F-box protein [17], [21], [26], [27], had EST redundancies ranging from 0.79% to 0.92%. One gene encoding a NAC family transcription factor had a high EST redundancy of 9.52%. Other genes with unknown functions also showed high EST redundancies of 10%–25% (Table 1). Interestingly, the majority of these unknown genes were derived from pepper and tomato plant clones.

**Table 1:** The unigene sequences representing proteins important for the ABA-regulated adaptive physiological processes to chilling stress.

Clone ID	Size (bp)	Accessionno.	Gene description	BLAST matching accession no.	Sequence identity (%)	*E* value	EST redundan-cy for a Gene (%)^a^
*Functional category-transcription*							
F007^b^	326	JZ198750	nam-like protein 4 (Petunia × hybrida)	AF509867.1	82	1e-28	9.52
F027^b^	292	JZ198770	MADS-box transcription factor (*C. annuum*)	DQ999998.1	85	1e-51	0.79
R028^b^	332	JZ198811	Multiprotein bridging factor 1 (*S. tuberosum*)	EU294363.1	80	1e-47	0.92
*Functional category-energy metabolism*							
F026^b^	295	JZ198769	carbonic anhydrase (*S. lycopersicum*)	NM001247119.1	91	2e-100	0.79
F035	309	JZ198778	alcohol dehydrogenase (*N. tabacum*)	X81853.1	86	3e-91	0.79
F039^b^	445	JZ198782	ribulose-1, 5-bisphosphate carboxylase/oxygenase small subunit (*C. annuum*)	AF065615.1	99	8e-165	0.79
R017	298	JZ198800	cytochrome P450 like_TBP (*N. tabacum*)	D64052.1	98	5e-51	0.92
*Functional category- metabolism*							
R011^b^	236	JZ198794	glyceraldehyde-3-phosphate dehydrogenase (*C. annuum*)	AJ246011.1	82	4e-25	0.92
R032^b^	296	JZ198815	o-diphenol-O-methyltransferase (*N.tabacum*)	X71430.1	92	3e-116	0.92
R029^b^	262	JZ198812	F-box protein (*Medicago truncatula*)	XM_003592058.1	75	1e-13	0.92
F038^b^	1110	JZ198781	Elongation Factor 2 (*Hordeum vulgare*)	AK362029.1	73	9e-74	0.79
*Functional category-defense response*							
F008^b^	311	JZ198751	chitin binding protein (*C. annuum*)	AF333790.1	90	1e-96	0.79
F031^b^	362	JZ198774	superoxidase dismutase (*S. lycopersicum*)	NM_001247840.1	78	2e-75	0.79
R018	311	JZ198801	NAD(P)H-dependent glutamate synthase (*Plasmodium yoelii*)	XM_718819.1	82	9e-04	0.92
R031^b^	261	JZ198814	dehydrin-like protein (*C. annuum*)	AY225438.1	84	4e-51	0.92
*Functional category-signal transduction*							
F037	273	JZ198780	serine/threonine kinase (*N. tabacum*)	DQ459385.1	85	6e-24	0.79
R030^b^	210	JZ198813	CBL-interacting serine/threonine-protein kinase 1 (CIPK1) (*A. thaliana*)	NM_202599.1	76	4e-06	0.92
*Functional category-transport*							
F030^b^	192	JZ198780	plasma membrane calcium ATPase (*A. thaliana*)	NM119927.2	76	1e-17	0.79
*Functional category-unknown function*							
F012^b^	219	JZ198755	*Capsicum annuum*	GU048903.1	83	5e-24	18.2
F029^b^	343	JZ198772	*Solanum lycopersicum*	AK322634.1	73	2e-43	10.3
R002^b^	1072	JZ198785	*Capsicum annuum*	GU048902.1	81	3e-54	10.09
R004^b^	513	JZ198787	*Capsicum annuum*	JF330775.1	71	4e-04	25.68

ESTs sequences from the forward and reverse SSH libraries of *C. annuum* were grouped into singletons and contigs using CAP3 assembly program and were termed as unigenes. The unigene sequences were blasted for homology search using blastn and blastx programmes at NCBI database. The search results for those unigenes representing proteins having regulatory function are summarized. EST redundancy of each unigene is also given along with the average size of the ESTs constituting the unigene. Unigenes from the forward SSH library were marked with ‘F00X’ and the corresponding unigenes from the reverse SSH library were marked with ‘R00X’.

a Percentage of the fraction of ESTs representing a unigene/total no. of ESTs.

b Clones selected for further qPCR analysis.

### 2.6 Confirmation of ABA-regulated differential gene expression under chilling stress

**Figure 4:**
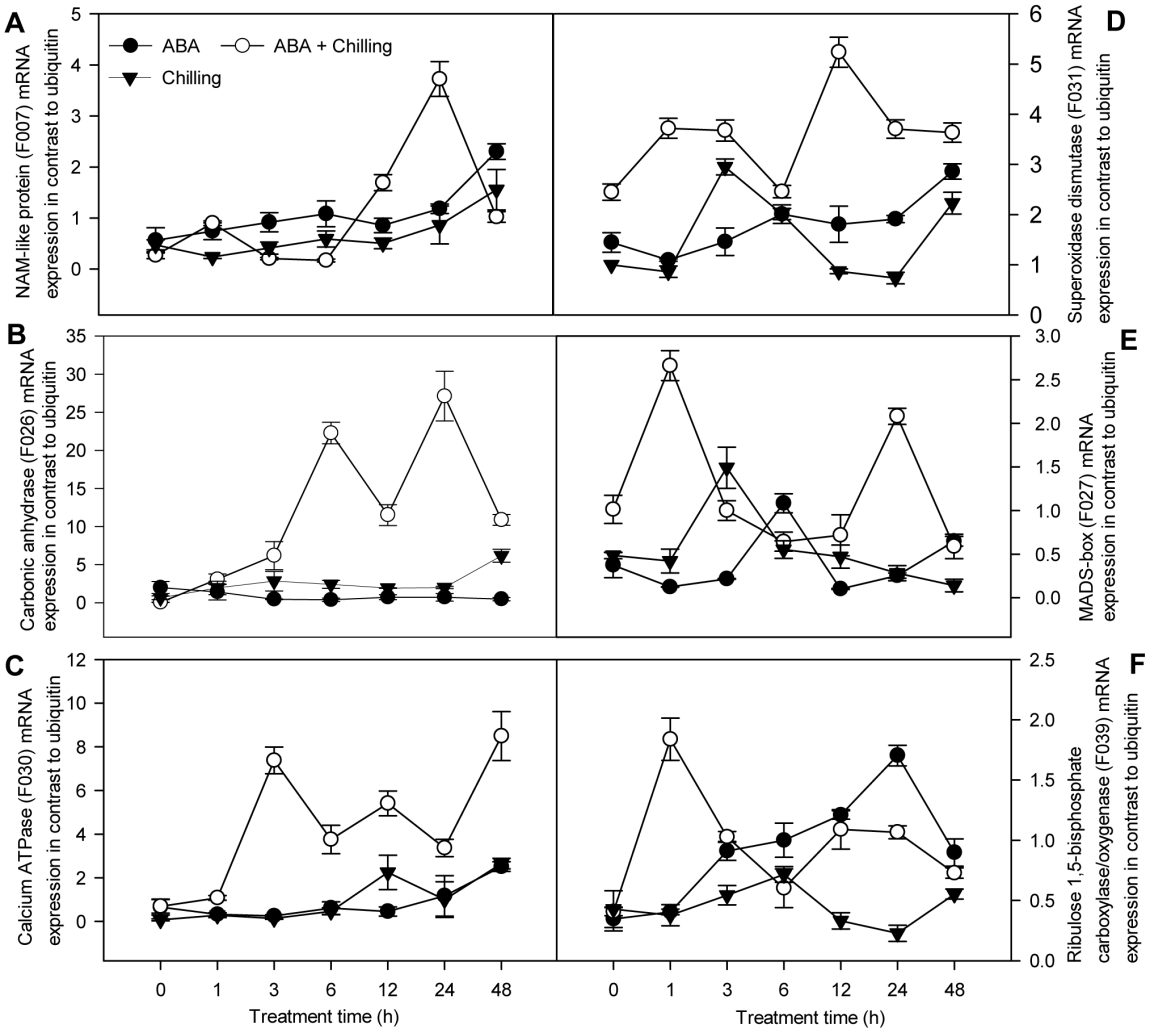
Analysis of mRNA expression of six genes using qPCR found upregulated with SSH. Total RNA was extracted from pepper leaves with ABA spray or water, subsequently 72 h after foliar application exposed for 48 h to 6°C. SE was calculated based on three biological replicates. Closed circle: ABA-pretreated samples under room temperature; open circle: ABA-sprayed samples prior to chilling stress; triangle down: water-sprayed samples prior to chilling stress. Reaction conditions for each gene are shown in ‘Materials and methods’. The level of ubiquitin was used as an internal reference gene.

**Figure 5:**
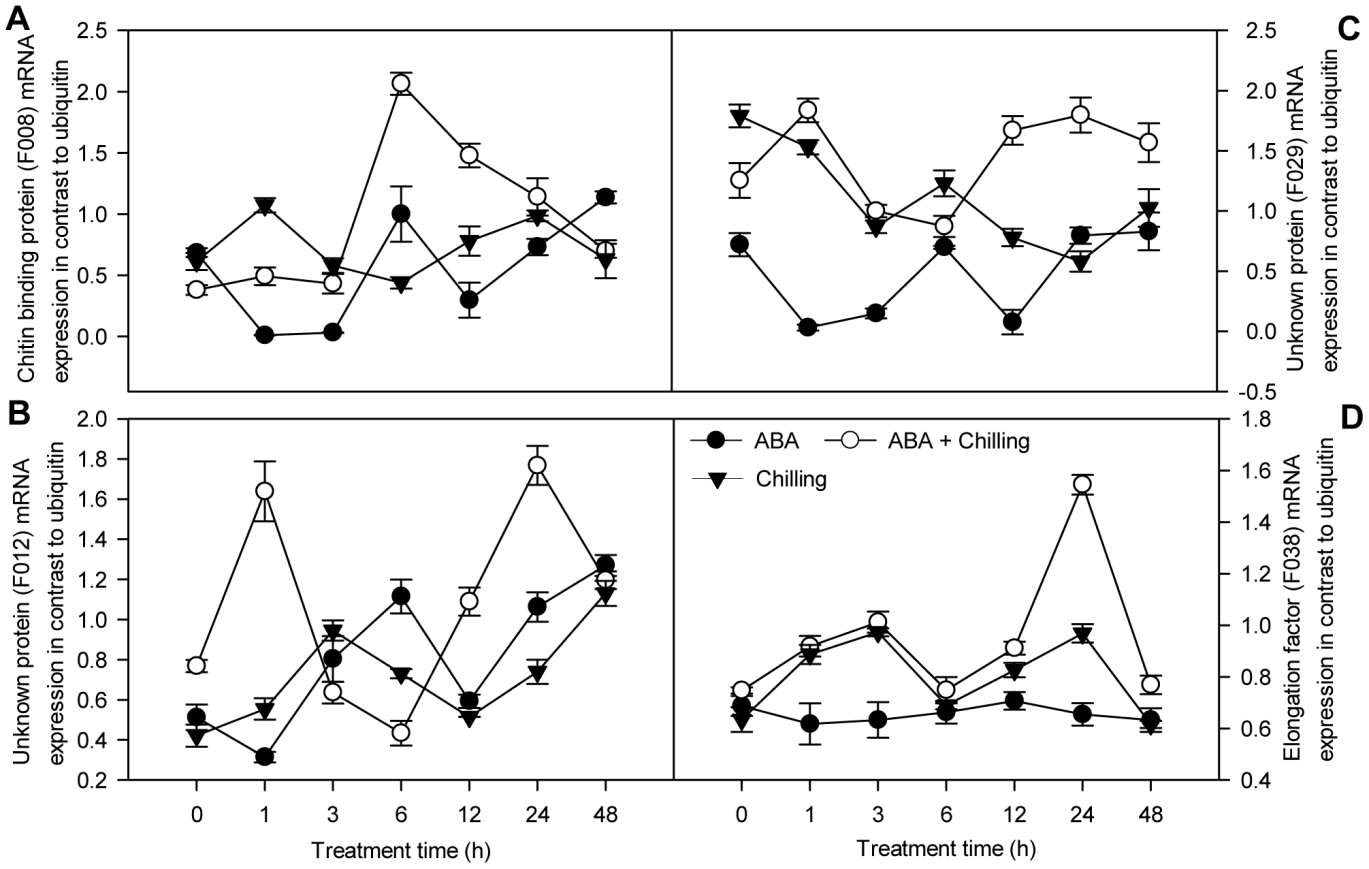
Analysis of mRNA expression of four genes using qPCR found upregulated with SSH. Total RNA was extracted from pepper leaves with ABA spray or water, subsequently 72 h after foliar application exposed for 48 h to 6°C. SE was calculated based on three biological replicates. Closed circle: ABA-pretreated samples under room temperature; open circle: ABA-sprayed samples prior to chilling stress; triangle down: water-sprayed samples prior to chilling stress. The level of ubiquitin was used as an internal reference gene.

To verify the genes identified from the forward and reverse SSH libraries were differentially expressed, we performed qPCR analysis to confirm the expression of 18 selected genes that were up- or downregulated by ABA treatment under chilling stress. The transcript levels of the 18 genes were investigated in ABA-pretreated and control pepper seedlings at different time points (0, 1, 3, 6, 12, 24 and 48 h) of chilling stress; the expression of these transcripts was also analyzed in ABA-pretreated seedlings under normal conditions at the same time points. All data was normalized to the ubiquitin gene. Figures 4 and 5 show the time courses for the transcript levels of the 18 genes selected from Table 1.

In this study, we designated genes with expression ratios (ABA+ chilling/control + chilling) of three-fold or greater at one or more points of the chilling stress time-course as ABA-upregulated genes under chilling stress. Similarly, genes showing expression ratios (ABA+ chilling/control + chilling) of one third or less were designated as ABA-downregulated genes under chilling stress. Genes with high expression levels prior to 6 h of chilling stress are likely to be involved in the early and transiently response to ABA under chilling stress, while genes showing high expression levels at 24 or 48 h of chilling stress were defined as late ABA-responsive genes under chilling stress; genes showing changes in expression both before 6 h of chilling stress and at 24 or 48 h of chilling stress were classified as early and continually ABA-responsive genes under chilling stress.

In the forward SSH library, the expression levels of four genes (F007, F026, F030 and F031; Fig. 4A–D) in ABA-pretreated seedlings subjected to chilling stress rapidly increased during early chilling stress (1 and 3 h) and reached the highest levels after extended chilling stress (at 12, 24 and 48 h). The expression levels of two genes (F027 and F039; Fig. 4E and 4F) were highest at 1 h chilling stress, slightly reduced at 6 h chilling stress, and increased again thereafter. Compared with control chilling-stressed seedlings, the expression ratios of these six genes (F007, F026, F030, F031, F027 and F039; Fig. 4) were increased by at least three-fold at both 1 or 6 h chilling stress and 24 or 48 h chilling stress, suggesting that these genes are early and continually ABA-upregulated genes under chilling stress. Of these six genes, F007 and F027 are a NAC-like protein and MADS-box transcription factor genes, respectively, and are implicated in transcription. F026 (encoding a carbonic anhydrase), F030 (a calcium ATPase gene) and F039 (a ribulose 1, 5-bisphosphate carboxylase/oxygenase or RuBisCo gene) are implicated in metabolism, and F031 (a superoxidase dismutase gene) is implicated in the defense response.

In addition, the expression ratios of the genes belonging to the early transiently ABA-upregulated genes under chilling stress (F008 and F012; Fig. 5A and 5B) and late ABA-upregulated genes under chilling stress (F029 and F038; Fig. 5C and D) increased by three-fold or more at 1 or 6 h chilling stress, or 24 h chilling stress, respectively. In contrast, the expression ratio of the putative elongation factor gene (F038) involved in metabolism increased by more than two-fold at 24 h chilling stress. The expression of all of these genes was higher in the ABA+ chilling treatment group than the control plants under chilling stress throughout the entire time course of chilling stress, suggesting that these genes are positively regulated by ABA under chilling stress. However, the expression levels of six genes (F026, F030, F027, F008, F029 and F038; Fig. 4B, 4C, 4E and Fig. 5A, 5C and 5D) did not alter in ABA-pretreated pepper seedlings under non-chilled conditions, but were upregulated in the ABA+ chilling treatment group during the time course, indicating that the ABA-inducible expression of these six genes under chilling stress was cold-dependent. The expression of F039 in ABA-pretreated seedlings under normal conditions (Fig. 4F) gradually increased to a higher level than that observed in the ABA+ chilling stress group. However, the expression of F039 did not significantly vary in response to chilling stress in control plants, indicating that the ABA-induced expression of this gene was not cold-dependent. Furthermore, the expression of F007 in only ABA-treated or chilling stressed seedlings gradually increased during the entire time course of chilling stress (Fig. 4A). In control pepper seedlings subjected to chilling stress, the expression of F030 gradually increased after 12 h chilling stress (Fig. 4C), whereas the expression of F038 rapidly increased at 1 h chilling stress, remained high (except at 6 h chilling stress), and recovered to the initial levels at 48 h chilling stress (Fig. 5D).

**Figure 6:**
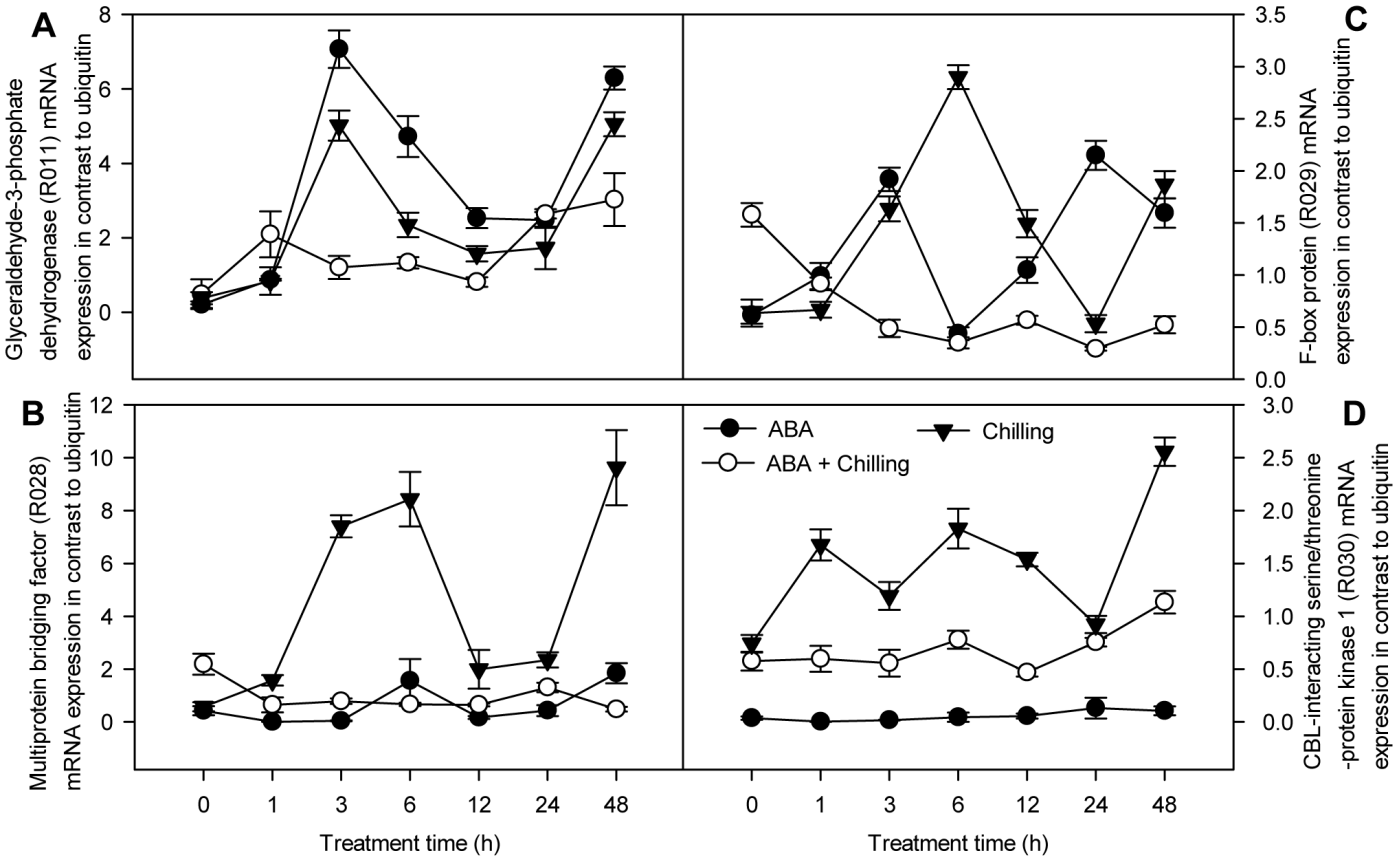
Analysis of mRNA expression using qPCR of four genes found downregulated with SSH. Total RNA was extracted from pepper leaves with ABA spray or water, subsequently 72 h after foliar application exposed for 48 h to 6°C. SE was calculated based on three biological replicates. Closed circle: ABA-pretreated samples under room temperature; open circle: ABA-sprayed samples prior to chilling stress; triangle down: water-sprayed samples prior to chilling stress. The level of ubiquitin was used as an internal reference gene.

**Figure 7:**
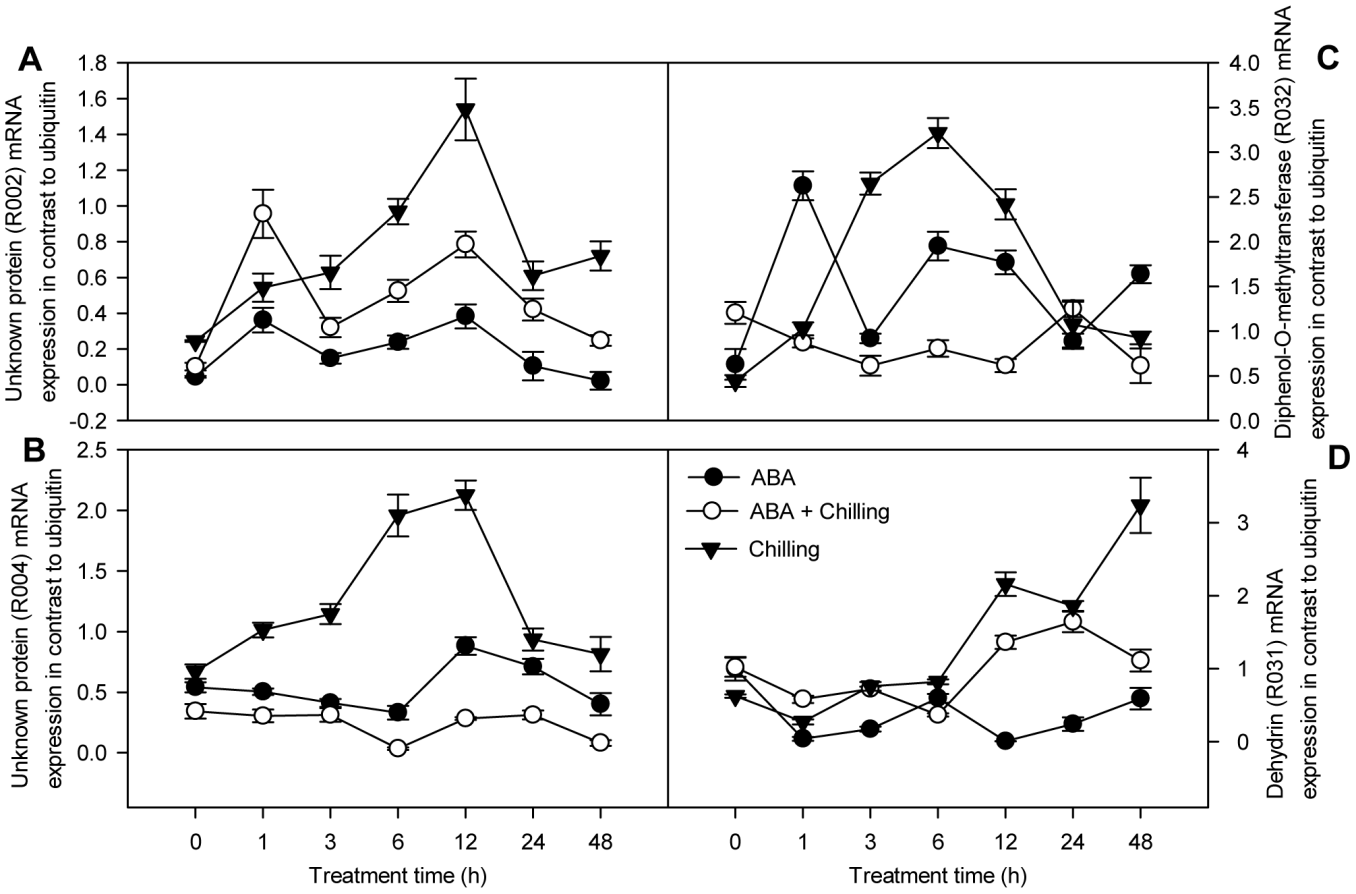
qPCR analysis of downregulated genes in the reverse SSH library. Total RNA was extracted from pepper leaves with ABA spray or water, subsequently 72 h after foliar application exposed to low temperature for 48 h to 6°C. SE was calculated based on three biological replicates. Closed circle: ABA-pretreated samples under room temperature; open circle: ABA-sprayed samples prior to chilling stress; triangle down: water-sprayed samples prior to chilling stress. The level of ubiquitin was used as an internal reference gene.

In the reverse SSH library, the transcript levels of four genes (R011, R028, R029 and R030; Fig. 6A–D) in control plants peaked at 3 or 6 h chilling stress, declined at 24 h chilling stress, and increased after 48 h chilling stress. The transcript levels of R002, R004 and R032 (Fig. 7A–C) gradually increased, peaked at 12 or 6 h chilling stress and declined thereafter. Expression of the dehydrin gene (R031; Fig. 7D), which is involved in the defense response, gradually increased during chilling, peaking after 48 h chilling stress. However, compared to control plants subjected to chilling, the ABA-pretreated seedlings showed reduced transcript levels the above-mentioned eight genes when exposed to chilling stress, with expression ratios (ABA+ chilling/control + chilling) one-third or less that of the control chilled plants at one or more points of time-course, indicating that these genes were downregulated by ABA under chilling stress. Moreover, reduced expression levels (less than one-third) of R028, R029 and R004 were observed at both 3 h chilling stress and 48 h chilling stress (Fig. 6B and 6C and Fig. 7B); therefore, these genes were classified as early continually downregulated genes. Furthermore, reduced expression levels (less than one-third) of R011, R030, R032 (Fig. 6A and 6D, Fig. 7C) and R002 and R031 (Fig. 7A and 7D) were observed at 1 or 3 h chilling stress, or 48 h chilling stress; therefore, these genes were classified as early and transiently downregulated genes, and late downregulated genes, respectively. Of these genes, R028 is involved in transcription, sharing sequence identity with multiprotein bridging factor (MBF), R030 is involved in signal transduction, sharing sequence identity with CBL-interacting serine/threonine-protein kinase 1 (CIPK1); and R011, R029 and R032 are involved in metabolism, sharing sequence identity with glyceraldehyde-3-phosphate dehydrogenase (GAPDH), F-box protein and diphenol-O-methyltransferase (OMT), respectively. In summary, the early and continually genes which were up- or downregulated in response to ABA under chilling stress are mainly involved in processes related to transcription or metabolism.

## Discussion

In this study, exogenous application of ABA alleviated cold damage in pepper seedlings (Fig. S1), consistent with the results of Guo et al. [21] who observed severe chilling injury symptoms on the non-pretreated pepper seedlings leaves, whereas the ABA-pretreated leaves were only slightly damaged. Thus, ABA-regulated gene products might play a role in stress tolerance. A large crosstalk exists between cold signaling and ABA signaling. In addition, the genes which were specifically up- or downregulated after ABA treatment in pepper seedlings subjected to chilling stress were different to the genes identified in previous studies based on ABA treatment alone in other plant species [17], [18]. The present study attempted to identify ABA-regulated genes associated with cold tolerance. Forward and reverse SSH libraries were generated, which led to the identification of 73 unigenes, with altered gene expression observed at every time-point of chilling stress. These genes may be of interest for further characterization of the physiological mechanisms underlying stress tolerance and the genetic engineering of stress-tolerant plants.

### 3.1 Electrolyte leakage, chlorophyll content, net photosynthetic rate and stomatal conductance analysis

Water loss is the most common visible symptom of low temperature stress in intact plant tissues, and eventually leads to wilting during exposure to low temperature. Wilting that occurs during and/or after low temperature exposure is attributed to a primary mechanism involving the loss of membrane properties or transition of the membranes from a normal fluid state to a restricted, less fluid, semi-crystalline state [28]. Increased electrolyte leakage under chilling stress is indicative of membrane damage [29] and electrolyte leakage was decreased by application of ABA (Fig. 1A), in line with the protective effect of ABA on the visible symptoms of chilling injury (Fig. S1). Similarly, other studies have shown that exogenous ABA treatment reduced electrolyte leakage under chilling stress [30], [31]. Loss of total chlorophyll content in non-treated pepper seedlings under chilling stress (Fig. 1B) was mitigated by ABA pretreatment, which correlates well with the results of previous studies [31]. The loss of total chlorophyll, which is possibly caused by ROS generated during chilling stress [32], is in line with our previous studies showing that chilling stress significantly increased the levels of malondialdehyde and hydrogen peroxide (H_2_O_2_) in pepper leaves [21]. Exogenous application of ABA also effectively alleviated the rapid reduction in the net photosynthetic rate (Pn) in the leaves of pepper seedlings under chilling stress (Fig. 1C), in accordance with the findings of Jiang et al. [33] in tomato plants. ABA also reduced stomatal conductance (g_s_) in pepper plants subjected to chilling stress for 3 h (Fig. 1D); this is the typical of the response to ABA treatment in many plants [34].

### 3.2 Genes with unknown functions

In total, 73 unigenes, which showed different levels of expression in the ABA-pretreated and non-treated pepper seedlings under chilling stress were identified, suggesting the involvement of a large number of genes in the ABA-regulated processes related to chilling tolerance. The contigs EST redundancy of 76.6% indicates the possibility of discovering of additional genes, particularly those encoding low abundance proteins, on continued cloning and sequencing of the forward and reverse SSH libraries. Furthermore, the 73 unigenes encode proteins involved in processes such as metabolism, defense, transcription, signal transduction, and other processes. The majority of the genes identified from the forward SSH library were associated with transcription (12.50%), indicating that ABA may regulate transcription by a variety of mechanisms in plants subjected to chilling.

Unigenes with unknown functions or low similarities to known genes were also relatively abundant. Approximately 30% of the unigenes in the two SSH libraries could not be functionally characterized. These functionally uncategorized ‘unknown or novel’ genes might be a potential source of ‘candidate’ abiotic-tolerant genes [35]. We identified the differential expression of four unknown genes with a high EST redundancy using qPCR. The expression levels of F012 and F029 were more than three-fold higher in ABA-pretreated seedlings subjected to chilling stress than control seedlings subjected to chilling stress at 1 h and 24 h, respectively (Fig. 5B and 5C), indicating that the ABA-responsive genes can be separately induced as either early or late responses to chilling stress. In addition, R002 and R004 were also strongly upregulated in control chilled seedlings compared to 0 h chilling stress (Fig. 7A and 7B), suggesting that these genes may be involved in the adaptation to chilling stress. Although the function of these genes in the ABA-regulated response to chilling stress remains unclear, these genes should be isolated and characterized as potentially novel genes related to chilling tolerance. It will be important to obtain the corresponding full-length genes and analyze their expression profiles during the response to chilling stress in the pepper.

### 3.3 Genes related to transport upregulated by ABA during chilling

Several lines of evidence suggest the involvement of Ca^2+^ pumps in salt tolerance, such as increased expression of plant endoplasmic reticulum (ER) Ca^2+^-ATPases in tomato [36] and tobacco [37]. In the present study, the transcript levels of a calcium-ATPase (F030) gradually increased in control pepper seedlings under chilling stress after 12 h (Fig. 4C), indicating that calcium-ATPase is involved in the late response to cold stress. ABA may mediate the effects of cold on stomatal aperture [38]. ABA binds to receptors on the external plasma membrane of stomatal guard cells and induces a signal transduction cascade involving increases in cytoplasmic calcium, which eventually reduces guard cell osmotic potential to cause stomatal closure [39]. The expression levels of the calcium ATPase also rapidly increased (Fig. 4C) in ABA-pretreated seedlings after 3 h of chilling stress (more than 5-fold compared to control chilled seedlings). This result, together with the observation that stomatal conductance abruptly reduced in ABA-pretreated seedlings subjected to chilling stress (Fig. 1D), indicates that the calcium ATPase may be involved in ABA-mediated stomatal closure under chilling stress. This suggests that the expression of plasma membrane calcium ATPase is regulated at the level of transcriptional, as has previously been suggested for stomatal guard cells [26], in addition to the well-documented regulation of calcium ATPase at the post-transcriptional level [40].

### 3.4 Genes related to transcription upregulated by ABA under chilling stress

Recent studies have shown that some ABA-inducible members of a plant-specific transcription factor family that contains a DNA binding NAC domain (no apical meristem [NAM], ATAF, and cup-shaped cotyledons [CUC]) are also responsible for ABA-mediated signal transduction responses. ANAC019, ANAC055, RD26/ANAC072, and ATAF1 were upregulated by ABA in *Arabidopsis* [41], [42]. In this study, the transcription factor NAC homologue (F007) was upregulated by ABA and cold treatments in pepper seedlings (Fig. 4A). The ABA-mediated drought stress signal induces NTL4 activity at both transcriptional and protein levels, and NTL4 promotes ROS production which is closely related to the drought-resistant response [43]. ABA-dependent dehydration signal upregulates RD26/ANAC072 expression, which is involved in ROS detoxification and defense [41]. In addition, overexpression of ANAC genes (ABA-responsive NACs) confers increased sensitivity to ABA and enhances stress tolerance in *Arabidopsis* [41], [44] and rice [45]. Notably, we found that a NAC transcription factor (F007) was strongly induced by ABA treatment in pepper seedlings subjected to chilling stress, with 4-fold increases observed compared to control plants at 1 and 24 h chilling stress (Fig. 4A). Considering that exogenous application of ABA may enhance the tolerance to chilling stress by reducing electrolyte leakage from the cell membrane (Fig. 1A), and decreasing the levels of malondialdehyde and H_2_O_2_ as reported in our previous studies [21], we hypothesize that the ABA-regulated NAC transcription factor activates a subset of genes that act together to enhance chilling tolerance; however, the precise details of this mechanism remain to be investigated.

The MADS-box genes encode a group of important transcription factors, which are involved in regulating the processes of development and signal transduction. Temperature-sensitivity is likely to be a common feature of many MADS-box genes [46], and at least 14 MADS-box genes are either up- or downregulated in response to cold stress in *Arabidopsis* [47]. In the present study, expression of a MADS-box gene (F027) rapidly peaked at 1 h in ABA-pretreated seedlings subjected to chilling stress, and this ABA-inducible expression was cold-dependent (Fig. 4E), indicating that the change of this gene transcript can be affected by cold temperature.

### 3.5 Genes related to metabolism and defense upregulated by ABA under chilling stress

A number of ESTs involved in protein synthesis and energy metabolism may be essential for the ABA-mediated stress tolerance responses to environmental stimuli in plants. Accumulating evidence indicates that components of the translational apparatus exert functions in cells beyond their conventional role in protein synthesis [48]. For example, a mutation in translation elongation factor 2 (EF2) specifically blocked the induction of downstream targets of EF2 under low temperature and also reduced the capacity of *Arabidopsis* plants to develop freezing tolerance [49]. The induction of EF2 transcript under chilling stress (F038; Fig. 5D) has also previously been reported in barley [50]. EF2 protein expression level is downregulated by ABA during seed dormancy breaking, where EF2 is responsible for protein synthesis and cell division in the root meristem [51]. In contrast, the expression of an EF2 homologue was activated by ABA at the transcriptional level in pepper seedlings subjected to chilling stress (Fig. 5D), indicating that this gene is involved in ABA-regulated cold tolerance.

Genes encoding enzymes related to photosynthesis (e.g., carbonic anhydrase, F026 and RuBisCo, F039) were also isolated from the forward SSH library. Carbonic anhydrase plays an important role in photosynthesis, possibly by affecting electron transfer and the supply of Rubisco with CO_2_ [52]. Transgenic rice carbonic anhydrase (OsCA1) *Arabidopsis* plants have a higher salt tolerance than wild-type plants at the seedling stage [53]. Expression of the carbonic anhydrase gene was rapidly induced by ABA treatment in pepper seedlings subjected to chilling stress (Fig. 4B); the role that this gene plays in chilling tolerance remains to be identified. Reduced activity of photosystem II during cold conditions results in ROS formation [54]. Furthermore, ABA reduces chilling-induced photoinhibition by protecting photosystem II [55], which may be related to the upregulation of a gene encoding small subunit of RuBisCo (Fig. 4F) and the attenuation of chlorophyll pigment loss observed in pepper leaves (Fig. 1B).

Several defense-related genes, such as superoxidase dismutase (SOD, F031) and chitin-binding protein (F008), were upregulated by ABA treatment in pepper seedlings subjected to chilling stress. The enzyme SOD efficiently dismutates O^2−^ into H_2_O_2_ in various cell organelles. Pretreating plants with ABA enhanced chilling tolerance by inducing expression of the SOD gene (Fig. 4D), which is involved in oxidative stress defense mechanisms, this observation correlates well with the results of previous studies [21]. The gene encoding chitin binding protein was also upregulated by ABA in pepper seedlings subjected to chilling stress (Fig. 5A). This gene has been implicated in biotic attack responses [56], suggesting that crosstalk exists between the signal transduction pathways induced by both biotic and abiotic stresses.

### 3.6 Genes downregulated by ABA under chilling stress

Analysis of genes which are downregulated and upregulated by ABA under chilling stress is important, in order to fully understand the molecular responses to abiotic stress. In the reverse SSH library, several regulation-related factors, including genes encoding a MBF transcription factor (R028), F-box protein (R029), and CIPK (R030), were downregulated after ABA treatment under chilling stress. F-box family proteins are key components of the ubiquitin/proteasome pathway, which mediates the selective degradation of regulatory proteins and plays an important role in plant abiotic stress tolerance [57]. F-box proteins are also key factors involved in phytohormone signaling [58]. In pepper seedlings, both ABA and chilling treatments upregulated the expression of putative F-box gene, and this result is supported by evidences from other researchers [57], [59]. A mutation in F-box protein (DOR) enhances substantial drought stress tolerance in *Arabidopsis* [27]. Furthermore, overexpression of an F-box protein gene reduced abiotic stress tolerance in rice [60]. It is likely that the ABA-mediated downregulation of F-box gene expression (Fig. 6C) aids in the cold tolerance of pepper leaves. CIPK1 is a major calcium signaling component in *Arabidopsis*, which is involved in salt and osmotic stress signaling and regulates ABA-dependent and ABA-independent stress responses [61]. Coactivators of MBF gene are a new class of transcription factors, which are capable of connecting a regulator DNA-binding protein and a component of the basal transcription machinery. In *Arabidopsis*, the MBF1 gene has been related to stress tolerance and plays a negative role in ABA-dependent inhibition of germination [62]. In the present study, the expression of the MBF and CIPK genes was induced by chilling stress in control plants (Fig. 6B and 6D) and was relatively downregulated in ABA-pretreated seedlings subjected to chilling stress, with expression ratios of 0.105-fold (at 3 h) and 0.31-fold (at 1 h) compared to control plants under chilling stress, respectively. These results suggest that CIPK and MBF may be involved in the rapid and flexible regulation of selective gene expression in pepper seedlings subjected to chilling stress.

A dehydrin gene (R031) was isolated from the reverse SSH library as a potential defense response gene. In *Arabidopsis*, different patterns of accumulation of dehydrins transcripts have been shown in response to low temperature and ABA [63]. Expression of the dehydrin gene increased in control seedlings in response to chilling stress (Fig. 7D), rather than ABA treatment. In addition, a metabolism-associated gene encoding a lignin biosynthetic protein (R032) was also identified in the reverse SSH library. Biosynthesis of lignin in plants is altered in responses to abiotic stress [64]. For example, increased expression of the gene encoding O-methyltransferase, which is involved in the biosynthesis of lignin and potentially flavonoid and phenylpropanoid conjugates, under drought stress [65] was associated with desiccation tolerance [66]. In the present study, expression of the OMT gene (R032) induced by only ABA treatment and chilling stress (Fig. 7C) might be involved in the regulation of lignin synthesis. Furthermore, another metabolism-associated gene, GAPDH (R011), was identified, which encodes a classical enzyme of the glycolysis pathway. However, recent studies revealed that GAPDH is a multifunctional protein involved in various cellular functions. For example, AtGAPC-1 directly interacts with H_2_O_2_, suggesting that this enzyme mediates ROS signaling in *Arabidopsis* [67]. Overexpression of OsGAPC3 improved the salt tolerance of transgenic rice plants via regulating of H_2_O_2_ levels [68]. Previous studies have shown that most plant GAPDH genes can be induced in responses to various stresses, such as cold stress in mushroom [69] and ABA in rice [68]. In the present study, expression of the GAPDH gene (R011) rapidly increased in pepper seedlings in response to both non-pretreated chilling stress and ABA treatment under normal conditions (Fig. 6A). In contrast, the expression of this gene was not dramatically altered in ABA-pretreated seedlings subjected to chilling stress. These results, together with observations in previous studies that ABA effectively reduces foliage damage and the levels of malondialdehyde and H_2_O_2_ under chilling stress [21], suggest that pretreatment with exogenous ABA was insufficient to activate GAPDH gene expression to prevent the over-accumulation of ROS under chilling stress.

The present study demonstrates that a number of stress-related genes were up- or downregulated by ABA in pepper plants subjected to chilling, compared to control chilled plants. However, a number of well-investigated stress-marker genes, such as RD29A, B, KIN1, KIN2, CBF1, RAB, were not isolated from the forward or reverse SSH libraries. This is probably due to the limited number of clones sequenced in this study; differences between the plant species tested; the intensity and duration of stress; and differences in the timing and methods of selection; and/or the presence of these genes in both the forward and reverse SSH libraries. Moreover, overlapping between the signaling pathways downstream of ABA and cold signaling have previously been identified and characterized [17], suggesting that the expression of stress-marker genes may not vary between the forward and reverse SSH libraries.

## Conclusion

In the present study, we focused on the ABA-regulated response of pepper plants to chilling stress, with the goal to analyze the expression profiles of ABA-mediated genes and ultimately identify ABA-regulated genes related to chilling stress. A total of 73 unigenes were identified to be up- or downregulated by ABA in pepper seedlings subjected to chilling stress. Of these 73 genes, only 37 genes could be classified into different functional categories based on their putative roles in defense. It is likely that those genes, which were up- or downregulated by exogenous ABA under chilling stress compared to control chilled plants, might have relevance, since ABA plays a pivotal role in many developmental processes and adaptive responses to environmental stimuli in plants. The information obtained in this study provides a valuable first step for further characterization of ABA-mediated candidate genes associated with chilling stress, as well as in-depth analyses of the relevance of several cold-tolerant mechanisms regulated by these gene products in both ABA-pretreated and control plants. It is not known whether ABA-regulated cold-related genes encode proteins, and the functions and targets of these genes have not yet been identified. Moreover, regulation of the expression and translation of these genes remains elusive. Further studies are in progress in our experiment to investigate the role of the 18 genes confirmed using qPCR. The involvement of NAC and MBF transcription factors in ABA-mediated gene expression should be investigated further, as they represent promising candidates for the development of useful transgenic crops through the appropriate temporal and spatial regulation of the expression of target genes related to chilling stress tolerance.

## Materials and Methods

### 5.1 Plant materials, ABA application and chilling stress treatment

Pepper (*Capsicum annuum* L.) cv. P70 seeds were sown in plastic pots after accelerated germination and grown in a growth chamber using a previously described method [21]. The plants were grown at 25±1°C/18±1°C (day/night) with a 12 h photoperiod, light intensity of 20,000 lx and relative humidity of 60–70% (day/night). When the plants had formed 6–8 leaves, the pots were divided into three groups: the control group, sprayed with distilled water; and two ABA treatment groups, sprayed with freshly prepared 0.57 mM ABA solution at 09:00 to 10:00 am, to completely wet both sides of the leaves. Three days after foliar application, the control group and one ABA treatment group were subjected to chilling stress at 6°C; the other ABA treatment group was maintained at room temperature with a 12 h photoperiod with a light intensity of 5,500 lx. Leaves were collected from both the ABA-treated and control plants at various time points after the start of chilling (0, 1, 3, 6, 12, 24 and 48 h). At each time point, two or three upper young leaves from four separate seedlings were collected to form one sample, wrapped with foil, immediately frozen in liquid nitrogen and stored at −80°C. The treatments were arranged in a randomized complete block design with four replicates.

### 5.2 Chlorophyll content determination

The total chlorophyll content was determined according to the method of Korkmaz et al. [4]. Leaves weighing 0.5 g were ground with 5 ml of acetone (80% v/v) and filtered through a filter paper (What-man No. 2). The absorbance was measured spectrophotometrically according to the formula proposed by Lichtenthaler (1987) [70]: Chl a+b (mg/g FW)  = 7.79×A _663_+16.26×A _645_. Each measurement was performed using fresh leaf samples from three randomly selected plants per replicate, and the experiments were repeated three times with similar results.

### 5.3 Electrolyte leakage

To assess membrane permeability, electrolyte leakage was determined according to the method described by Dionisio-Sese and Tobita [71]. Briefly, ten leaf discs (1 cm in diameter) were obtained from the upper, fully expanded youngest leaves of two randomly chosen plants per replicate and washed with distilled water to remove surface contamination. The discs were placed individually in test tubes containing 10 ml of distilled water, incubated in a water bath at room temperature for 2 h, and the initial electrical conductivity (EC1) of the medium was measured using an electrical conductivity analyzer (DDS-307; Shanghai Precision Scientific Instrument Co., Ltd., China). The samples were autoclaved at 100°C for 30 min to release all of the electrolytes before cooling to 25°C to obtain the final electrical conductivity (EC2). Electrolyte leakage was calculated as EC1/EC2 and expressed as a percentage.

### 5.4 Net photosynthetic rate and stomatal conductance analysis

The net photosynthetic rate (P_n_) and stomatal conductance (g_s_) were measured using a LI-6400XT Portable Photosynthesis System (Li-Cor Inc., Lincoln, NE, USA). Leaves were selected from the same position on the plants (third or fourth from the shoot apex), with five replicates each from both control and ABA-treated plants. The conditions inside the leaf chamber were the same as the growth chambers: 6°C, 70% RH, photosynthetically active radiation (PAR)  = 100 µmol m^−2^ s^−1^, and CO_2_  = 520 µmol mol^−1^.

### 5.5 RNA isolation and cDNA preparation

Total RNA was isolated from the leaves of pepper plants sprayed with ABA or distilled water using TRIZOL reagent (Invitrogen Carlsbad, CA, USA) according to the manufacturer's instructions. For SSH library construction, the ABA-pretreated samples for ‘P70’ cv. pepper plants were generated by combining equimolar RNA from the chilling-stressed leaves harvested at the 0, 1, 3, 6, 12, 24 and 48 h time points. In a parallel scheme, the control samples of ‘P70’ cv. pepper plants were created by merging RNA from chilling-stressed leaves at each time point. Poly (A) + mRNA was isolated using the PolyATtract® mRNA Isolation Systems IV (Promega, USA) following the manufacturer's instructions. The concentrations of total RNA and mRNA were measured spectrophotometrically using a NanoDrop instrument (Thermo Scientific NanoDrop 2000C Technologies, Wilmington, USA), and the purity was assessed using the A260/280 and A260/230 ratios and electrophoresis on 1.0% agarose gels followed by ethidium bromide staining according to Gao et al. [72].

### 5.6 Construction of SSH cDNA libraries

The forward and reverse SSH cDNA libraries were prepared using the PCR select–cDNA SSH kit (Clontech, Palo Alto, USA) according to the manufacturer's instructions. The cDNAs from control (water-pretreated) and ABA-pretreated plants under chilling stress were digested with *Rsa*I at 37°C for 1.5 h, extracted with phenol/chloroform, precipitated with ethanol, and resuspended in water. The *Rsa*I -digested cDNAs of the water control (C) and ABA-pretreated (T) samples under chilling stress were divided into four equal parts. One part each of the C and T cDNA populations were separately ligated to adapter-1 (supplied in the SSH kit) at 16°C overnight; the ligated products were called CA1 (*Rsa*I-digested cDNA population from control sample subjected to chilling stress with adapter-1) and TA1 (*Rsa*I -digested cDNA population from ABA-pretreated samples under chilling stress with adapter-1), respectively. Another part each of the C and T cDNA populations were ligated to adaptor-2R in a similar fashion, and called C2R and T2R, respectively. The remaining two parts of both C and T, representing the *Rsa*I-digested blunt ended cDNAs of the control and ABA-pretreated samples under chilling stress, respectively, were used as the “Drivers”.

To construct the forward SSH library, representing an enriched population of the overexpressed and newly-induced transcripts, TA1 and T2R were used as “Tester-A” and “Tester-B”, respectively, and the C as the “Driver”. CA1 and C2R were considered as “Tester-A” and “Tester-B”, respectively, and the T as the “Driver” for the creation of the reverse SSH library representing an enriched population of downregulated transcripts. Two rounds of hybridization were performed. The Driver cDNA was added to each of the tester samples, which were subsequently resuspended in hybridization buffer. After heat-denaturation, the mixtures were allowed to anneal at 68°C for 8 h. Subsequently, two samples from the first hybridization were mixed together, freshly denatured driver cDNA was added to the sample followed by incubation at 68°C overnight for the second hybridization. Two rounds of PCR amplification using two different nested primers were conducted to amplify the differentially expressed cDNAs. The secondary PCR products of the forward and reverse SSH cDNA libraries were directly cloned into the pGEM–T easy vector (Promega, WI, USA) and transformed into *E. coli* DH5α (Invitrogen, CA, USA). The recombinant clones were plated onto LB medium containing ampicillin, X–Gal and IPTG and incubated overnight at 37°C for blue-white colony screening. All white clones were selected to construct the subtracted libraries.

### 5.7 Amplification of cDNA inserts

96 white clones were randomly picked from the forward and reverse SSH libraries and cultured overnight in 96-well plates in 200 µL LB/ampicillin broth at 37°C. To estimate the range of cDNA inserts from these clones, PCR amplification was employed using M13 primers (forward: 5′–GTAAAACGACG GCCAG–3′, reverse: 5′–CAGGAAACAGCTATGAC–3′) in 25 µL PCR mixtures containing 1.5 µL bacterial culture (template), 2.5 µL 10×PCR buffer, 1.0 µL of each primer (10 mM each), 2.0 µL dNTPs mix (2.5 mM each), 16.7 µL sterile water and 0.3 µL (2.5 UµL^−1^) of *Taq* DNA polymerase (TaKaRa Biotechnology, Dalian, China). The PCR reactions were performed using the following parameters: an initial denaturation at 94°C for 3 min, followed by 35 cycles of 94°C for 40 s, 55°C for 40 s, 72°C for 1.5 min, with a final extension at 72°C for 10 min. The PCR products were assessed using 1.0% agarose gel electrophoresis. Colonies containing inserted fragments with a single band over 300 bp were sequenced.

### 5.8 Sequencing and analysis of the cloned ESTs

The sequences obtained were screened to remove the vector, adaptor and poor quality sequences using the National Center of Biotechnology Information (NCBI) UniVec database (NCBI VecScreen, The UniVec Database 2010). Subsequently, ESTs longer than 100 bp were considered for further analysis. CAP3 software [73] was used to assemble the individual EST into unigenes, including contigs and singlets with a minimum overlap of 40 bp and minimum percentage identity of 97%. The resulting pepper unigene sequences were compared with the non-redundant (nr) database in GenBank using the blastx and blastn programs. cDNAs with a threshold *E*-value of 1^-03^ were designated as significant homology. The putative physiological functional categories of the identified unigenes were assigned based on GoFigure of the Gene Ontology annotations (http://www. geneontology.org).

### 5.9 qPCR analysis

Total RNA was extracted from the leaves of pepper plants treated with ABA or distilled water and subsequently subjected to chilling for 0, 1, 3, 6, 12, 24, and 48 h as described above. To validate the results of the SSH experiment, qPCR amplification of 18 selected genes was performed using specific oligonucleotide primers (Table S2) designed by Primer Premier 5.0. First-strand cDNA synthesis was performed using the PrimeScript™ first strand complementary DNA (cDNA) Synthesis Kit (TaKaRa, Japan), and qPCR was performed in triplicate in 96-well plates using SYBR® Premix Ex *Taq*™ II (TaKaRa, Japan) on an IQ5 real-time PCR instrument (Bio-Rad, Hercules, CA, USA). Each reaction (20 µL) consisted of 10 µL SYBR® Premix Ex *Taq™* II, 2 µL diluted cDNA, and 0.4 µM forward and reverse primers. The following qPCR cycling conditions were used: 95°C for 1 min, followed by 45 cycles of 95°C for 15 s, 54°C for 20 s, and 72°C for 30 s. The fluorescence data was collected during the 54°C step. The ubiquitin-conjugating protein gene (UBI-3, GenBank accession no. AY486137.1) from pepper plants was amplified using the primers (forward: 5′-TGTCCATCTGCTCTCTGTTG-3′, reverse: 5′-CACCCCAAGCACAATAAGAC- 3′) as a reference gene for normalization of the expression levels of the selected genes [74]. The analysis was performed with IQ5 software using the normalized-expression method.

## Supporting Information

Figure S1 **Effect of ABA application on visible chilling injury symptoms of pepper seedlings.** A, pretreated with 0.57 mM ABA and chilling stress for 1 h; B, pretreated with water and chilling stress for 1 h; C, pretreated with 0.57 mM ABA and chilling stress for 3 h; D, pretreated with water and chilling stress for 3 h; E, pretreated with 0.57 mM ABA and chilling stress for 6 h; F, pretreated with water and chilling stress for 6 h; G, pretreated with 0.57 mM ABA and chilling stress for 12 h; H, pretreated with water and chilling stress for 12 h; I, pretreated with 0.57 mM ABA and chilling stress for 24 h; J, pretreated with water and chilling stress for 24 h; K, pretreated with 0.57 mM ABA and chilling stress for 48 h; L, pretreated with water and chilling stress for 48 h; The differences among treatments are marked with white arrows in leaves. Photographs show plants subjected to chilling stress after foliar application 72 h.
(TIF)

Figure S2 **Total RNA of leaves in pepper seedlings.**
(TIF)

Figure S3 **mRNA isolated from pepper seedlings subjected to chilling stress.** Lane 1, mRNA from ABA-pretreated leaves under chilling stress; Lane 2, mRNA from water-pretreated leaves under chilling stress; M: DNA size marker.
(TIF)

Figure S4 **Results of SSH using leaves as testers and drivers.** This shows the secondary PCR products. Lane 1, reverse-unsubtracted leaves cDNA; lane 2, forward-unsubtracted leaves cDNA; lane 3, reverse-subtracted leaves cDNA; lane 4, forward-subtracted leaves cDNA. M: DNA size marker.
(TIF)

Figure S5 **A few inserts of subtracted cDNA clones of leaves amplified by PCR.** Lanes 1–7 show 7clones randomly selected from the subtracted library. M: DNA size marker.
(TIF)

Table S1 **The accession numbers of ABA-regulated unigenes in pepper plants under chilling stress.**
(DOC)

Table S2 **Primer sequences for qPCR.**
(DOC)
