# Supplementary figures and images for: Suppression Subtractive Hybridization Analysis of Genes Regulated by Application of Exogenous Abscisic Acid in Pepper Plant (Capsicum annuum L.) Leaves under Chilling Stress
Source: PLoS One. 2013 Jun 18;8(6):e66667. doi: 10.1371/journal.pone.0066667 (PMC3688960; doi:10.1371/journal.pone.0066667)

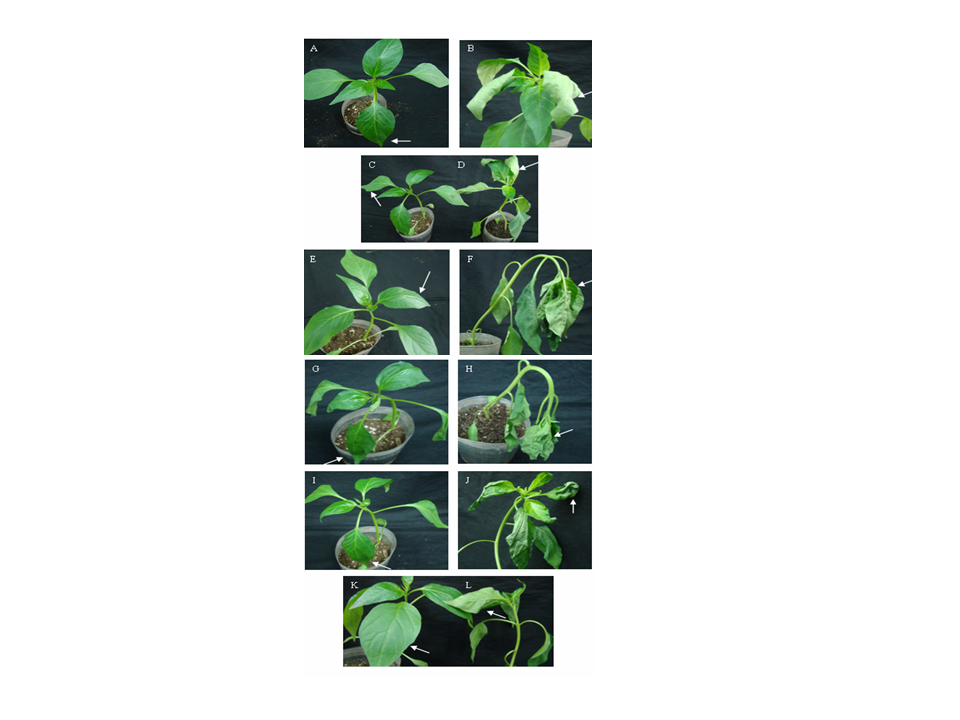

Supplement: Figure S1 — Effect of ABA application on visible chilling injury symptoms of pepper seedlings. A, pretreated with 0.57 mM ABA and chilling stress for 1 h; B, pretreated with water and chilling stress for 1 h; C, pretreated with 0.57 mM ABA and chilling stress for 3 h; D, pretreated with water and chilling stress for 3 h; E, pretreated with 0.57 mM ABA and chilling stress for 6 h; F, pretreated with water and chilling stress for 6 h; G, pretreated with 0.57 mM ABA and chilling stress for 12 h; H, pretreated with water and chilling stress for 12 h; I, pretreated with 0.57 mM ABA and chilling stress for 24 h; J, pretreated with water and chilling stress for 24 h; K, pretreated with 0.57 mM ABA and chilling stress for 48 h; L, pretreated with water and chilling stress for 48 h; The differences among treatments are marked with white arrows in leaves. Photographs show plants subjected to chilling stress after foliar application 72 h. (TIF) [file pone.0066667.s001.tif]
