# Supplementary material for: Suppression Subtractive Hybridization Analysis of Genes Regulated by Application of Exogenous Abscisic Acid in Pepper Plant (Capsicum annuum L.) Leaves under Chilling Stress
Source: PLoS One. 2013 Jun 18;8(6):e66667. doi: 10.1371/journal.pone.0066667 (PMC3688960; doi:10.1371/journal.pone.0066667)
Supplement: Table S1 — The accession numbers of ABA-regulated unigenes in pepper plants under chilling stress. (DOC) [file pone.0066667.s006.doc]

**Table S1. The accession numbers of ABA-regulated unigenes in pepper plants under chilling stress.**

| **Clone ID** | **Accession no.** | **Clone ID** | **Accession no.** | **Clone ID** | **Accession no.** |
| --- | --- | --- | --- | --- | --- |
| F001 | JZ198744 | F002 | JZ198745 | F003 | JZ198746 |
| F004 | JZ198747 | F005 | JZ198748 | F006 | JZ198749 |
| F007 | JZ198750 | F008 | JZ198751 | F009 | JZ198752 |
| F010 | JZ198753 | F011 | JZ198754 | F012 | JZ198755 |
| F013 | JZ198756 | F014 | JZ198757 | F015 | JZ198758 |
| F016 | JZ198759 | F017 | JZ198760 | F018 | JZ198761 |
| F019 | JZ198762 | F020 | JZ198763 | F021 | JZ198764 |
| F022 | JZ198765 | F023 | JZ198766 | F024 | JZ198767 |
| F025 | JZ198768 | F026 | JZ198769 | F027 | JZ198770 |
| F028 | JZ198771 | F029 | JZ198772 | F030 | JZ198773 |
| F031 | JZ198774 | F032 | JZ198775 | F033 | JZ198776 |
| F034 | JZ198777 | F035 | JZ198778 | F036 | JZ198779 |
| F037 | JZ198780 | F038 | JZ198781 | F039 | JZ198782 |
| F040 | JZ198783 | R001 | JZ198784 | R002 | JZ198785 |
| R003 | JZ198786 | R004 | JZ198787 | R005 | JZ198788 |
| R006 | JZ198789 | R007 | JZ198790 | R008 | JZ198791 |
| R009 | JZ198792 | R010 | JZ198793 | R011 | JZ198794 |
| R012 | JZ198795 | R013 | JZ198796 | R014 | JZ198797 |
| R015 | JZ198798 | R016 | JZ198799 | R017 | JZ198800 |
| R018 | JZ198801 | R019 | JZ198802 | R020 | JZ198803 |
| R021 | JZ198804 | R022 | JZ198805 | R023 | JZ198806 |
| R024 | JZ198807 | R025 | JZ198808 | R026 | JZ198809 |
| R027 | JZ198810 | R028 | JZ198811 | R029 | JZ198812 |
| R030 | JZ198813 | R031 | JZ198814 | R032 | JZ198815 |
| R033 | JZ198816 |  |  |  |  |
