# Supplementary material for: Suppression Subtractive Hybridization Analysis of Genes Regulated by Application of Exogenous Abscisic Acid in Pepper Plant (Capsicum annuum L.) Leaves under Chilling Stress
Source: PLoS One. 2013 Jun 18;8(6):e66667. doi: 10.1371/journal.pone.0066667 (PMC3688960; doi:10.1371/journal.pone.0066667)
Supplement: Table S2 — Primer sequences for qPCR. (DOC) [file pone.0066667.s007.doc]

**Table S2. Primer sequences for real-time PCR.**

| **Clone ID** | **Forward primer (5’-3’)** | **Reverse primer (5’-3’)** |
| --- | --- | --- |
| F007 | CGTTTTCAGGTCCCCTCTAT | CGAGGTCTTCCTTTTGTTTG |
| F008 | GGTACGACTAGGGAATACTGTG | TATCATACGTTTATTAGTTGGGAC |
| F012 | CCTTACTTATGATACCGTGGATT | TACTCTGCGTTGATACCACTG |
| F026 | TTGTGAGGGCCAAATGGT | TGGCATTGAAGGGAGGTTA |
| F27 | AGGAACCCAACCATCAGCA | TTTGAAAGGCAACACCAAGT |
| F029 | ACGCAGAGTTGAACCGAGAA | GTCCTTTCCATGACTTTTCG |
| F030 | ATGTATGATCTACTGCCTGGTG | CATTACTGGCTCACTTTCTCC |
| F031 | ATGTTTGACCCATTATGCTGA | ATACCCATGAAGTCCACGAG |
| F038 | GAAACCTATAAAAAGGGCGC | TTTATAGACTCGTCGGTGTTG |
| F039 | ATGAAGTCCAGGAGGCAAAG | TCTCATAAACTCAACTGGAATCC |
| R002 | TCCGAGATGAAGGGAAAGC | CTGGAACGCACAGATGACC |
| R004 | CATTACGAGGGGTGAGTTG | TAAGATGGGGATGACTAAGCT |
| R011 | TTGATGCTCTGTTGCTGTTAA | TTCTCGTACCTTAACCTATGACTTC |
| R028 | TACATCTATGAACACCAGGACAC | TGTAACTTTCAGTGCAGCATC |
| R029 | CCATGAAGCCGTTAGAGGAT | ACGAATAGATGCGTTAGTCAA |
| R030 | ATGAAGAATCCACATGGCAGAA | GCAATCATCGCGTTAAAATG |
| R031 | AGTGATCATTCTTTGCTTTATTC | TTAACTTTCTCACCAAACTCAGA |
| R032 | ACATCTAAATACCCCACAATTAAG | GTGACTATCACTCCAGTCATGAAG |
